# Hsp90 modulates the stability of MLKL and is required for TNF-induced necroptosis

**DOI:** 10.1038/cddis.2015.390

**Published:** 2016-02-11

**Authors:** X M Zhao, Z Chen, J B Zhao, P P Zhang, Y F Pu, S H Jiang, J J Hou, Y M Cui, X L Jia, S Q Zhang

**Affiliations:** 1 State Key Laboratory of Cellular Stress Biology, Innovation Center for Cell Signaling Network, School of Life Sciences, Xiamen University, Xiamen, Fujian, China

## Abstract

The pseudokinase mixed lineage kinase domain-like protein (MLKL) is a key component of tumor necrosis factor (TNF)-induced necroptosis and plays a crucial role in necroptosis execution. However, the mechanisms that control MLKL activity are not completely understood. Here, we identify the molecular chaperone Hsp90 as a novel MLKL-interacting protein. We show that Hsp90 associates with MLKL and is required for MLKL stability. Moreover, we find that Hsp90 also regulates the stability of the upstream RIP3 kinase. Interference with Hsp90 function with the 17AAG inhibitor destabilizes MLKL and RIP3, resulting in their degradation by the proteasome pathway. Furthermore, we find that Hsp90 is required for TNF-stimulated necrosome assembly. Disruption of Hsp90 function prevents necrosome formation and strongly reduces MLKL phosphorylation and inhibits TNF-induced necroptosis. Consistent with a positive role of Hsp90 in necroptosis, coexpression of Hsp90 increases MLKL oligomerization and plasma membrane translocation and enhances MLKL-mediated necroptosis. Our findings demonstrate that an efficient necrotic response requires a functional Hsp90.

Necrosis has long been viewed as an accidental and uncontrolled type of cell death. However, accumulating evidence has now clearly demonstrated that necrosis, like apoptosis, can be a regulated and programmed process. One specific form of programmed necrosis that is triggered by death receptors when caspases are inhibited has been termed necroptosis.^1^ In the past several years, necroptosis has emerged as a major alternative form of programmed cell death that has important roles in development, host defense against viral infection and inflammation.^1^

The receptor-interacting serine-threonine kinase RIP1 and its family member RIP3 have crucial roles in tumor necrosis factor (TNF)-induced necroptosis.^2, 3, 4, 5, 6^ Upon TNF stimulation, RIP1 binds to RIP3 via the RIP homotypic interaction motif.^4, 5, 6^ RIP3 then recruits the pseudokinase mixed lineage kinase domain-like protein (MLKL) to form a multiprotein complex referred to as the ‘necrosome' to trigger necroptosis.^7, 8^ MLKL binds to RIP3 through its pseudokinase domain. The phosphorylation of RIP3 at Ser227 is required for MLKL binding.^7^ Within the necrosome, RIP3 phosphorylates MLKL at Thr357 and Ser358.^7^ These phosphorylation events induce a conformational change in MLKL, which leads to MLKL activation.^9^ Upon activation, MLKL forms oligomers and translocates to the plasma membrane to execute necrotic cell death through a mechanism not well understood.^10, 11, 12, 13, 14, 15^

MLKL consists of two domains, the N-terminal helical domain (amino acids 1–178), and the C-terminal pseudokinase domain (amino acids 179–471).^7, 9^ The N-terminal region is the death effector domain. The pseudokinase domain is a regulatory domain, which is responsible for binding to the upstream RIP3 kinase. The indispensable role of MLKL in necroptosis has been demonstrated in MLKL^−/−^ mice.^9, 16^

Despite MLKL has an important role in necroptosis, little is known about its regulation. Here, we report the identification of the molecular chaperone heat shock protein 90 (Hsp90) as a novel MLKL-interacting protein using yeast two-hybrid screen. We show that Hsp90 is required for MLKL and RIP3 stability. Disruption of Hsp90 function prevents necrosome assembly and inhibits TNF-induced necroptosis. Our results suggest that Hsp90 has an important role in TNF-triggered necroptosis.

## Results

### Identification of Hsp90 as an MLKL-interacting protein

**Figure 1:**
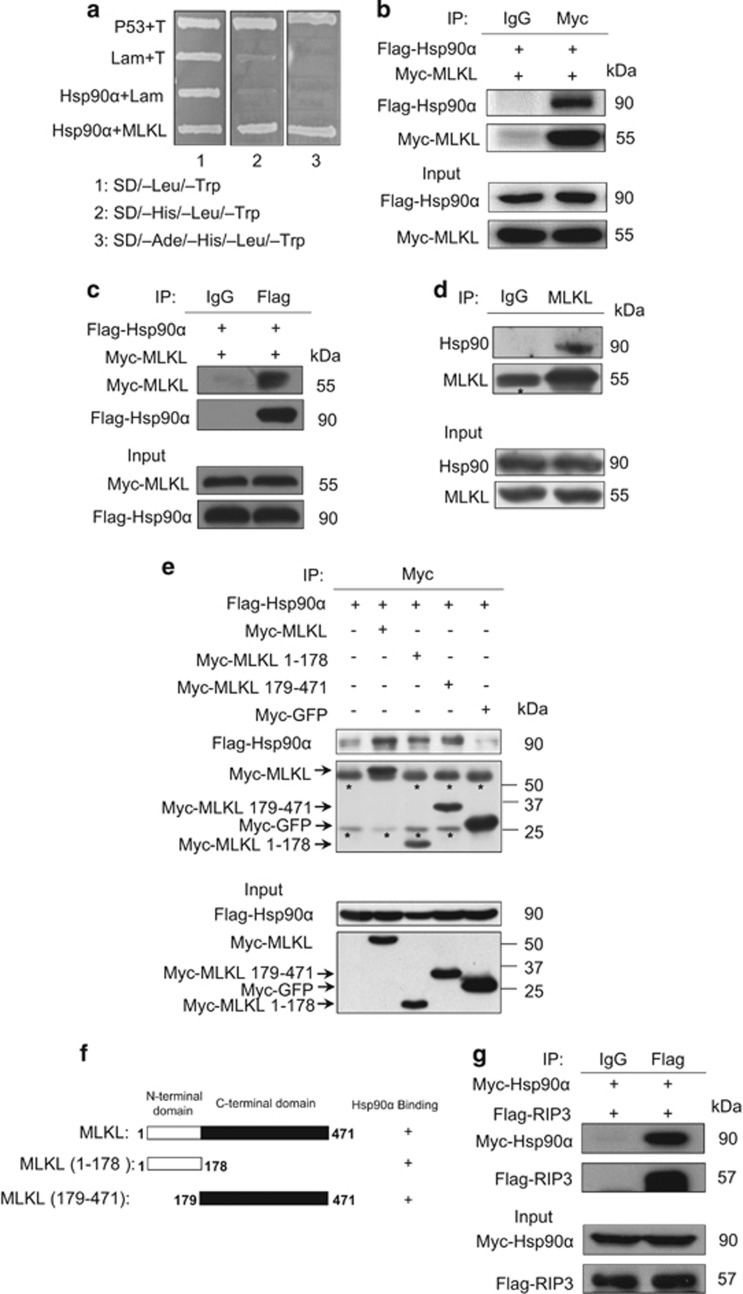
Hsp90 interacts with MLKL and RIP3. (**a**) Hsp90*α* interacts with MLKL in a yeast two-hybrid assay. AH109 yeast cells were co-transformed with the pACT2-Hsp90*α* (642–732aa) and pGBKT7-MLKL (1–178aa) plasmids and then plated on SD/-Leu/-Trp, SD/-His/-Leu/-Trp or SD/-Ade/-His/-Leu/-Trp selection media to allow growing for 1 week. Positive control pGBKT7-p53+pGADT7-T, negative control pGBKT7-Lam+pGADT7-T. (**b**) Flag-Hsp90*α* interacts with Myc-MLKL *in vivo*. The HEK293T cells were co-transfected with Flag-Hsp90*α* and Myc-MLKL, and cell lysates were immunoprecipitated with the indicated antibodies and immunoblotted with anti-Flag or anti-Myc antibody. (**c**) Myc-MLKL binds to Flag-Hsp90*α*. The 293T cells were co-transfected with Myc-MLKL and Flag- Hsp90*α*, and whole-cell lysates were immunoprecipitated with the indicated antibodies and subsequently analyzed by western blotting for the indicated proteins. (**d**) Endogenous Hsp90 associates with MLKL. Human Hela cervical carcinoma cell line whole-cell lysates were immunoprecipitated with the indicated antibodies and subsequently probed with anti-Hsp90 or anti-MLKL antibody. The asterisk denotes nonspecific IgG band. (**e**) Mapping of the MLKL domains responsible for binding to Hsp90*α*. Myc-MLKL or the indicated Myc-MLKL fragments and Myc-GFP were co-transfected with Flag-Hsp90*α* into 293T cells. After 24 h of transfection, cell lysates were immunoprecipitated with the indicated antibodies before western blotting. The asterisks denote nonspecific IgG bands. (**f**) Schematic representations of WT MLKL and the deletion mutants of MLKL used in the experiment. (**g**) Flag-RIP3 associates with Myc-Hsp90*α in vivo*. The 293T cells were co-transfected with Flag-RIP3 and Myc-Hsp90*α*, and cell lysates were immunoprecipitated with the indicated antibodies and probed with anti-Myc or anti-Flag antibody

To better understand the regulation of MLKL, we sought to identify MLKL-interacting proteins using a yeast two-hybrid screen. We screened a human bone marrow cDNA library with MLKL (1–178aa.) as bait. The screen identified a clone encoding Hsp90, as confirmed by sequencing, to be a novel MLKL-interacting protein (Figure 1a). To confirm the MLKL-Hsp90 interaction in mammalian cells, 293T cells were transfected with Flag-tagged Hsp90*α* and Myc-tagged MLKL. Extracts from the transfected cells were subjected to immunoprecipitation with an anti-Myc antibody, and the immunoprecipitates were probed with anti-Flag antibody for the presence of Flag-Hsp90*α*. As shown in Figure 1b, Flag-Hsp90*α* was readily detected in the Myc-MLKL immunoprecipitates but not in the control immunoprecipitates. In a reciprocal coIP experiment, Myc-MLKL can be readily detected in the Flag-Hsp90*α* immunoprecipitates (Figure 1c). We also observed that endogenous Hsp90 associates with endogenous MLKL in Hela cells (Figure 1d). These results demonstrate that Hsp90 physically interacts with MLKL in cells.

To determine the MLKL region involved in the interaction with Hsp90, Myc-tagged full-length MLKL and two MLKL deletion mutants were coexpressed with Flag-Hsp90*α* in 293T cells. A coimmunoprecipitation assay was performed with anti-Myc antibody. As shown in Figure 1e, both Myc-MLKL (1–178) and Myc-MLKL (179–471) co-precipitated with Flag-Hsp90*α*. The binding of Myc-MLKL (1–178) to Flag-Hsp90*α* in 293T cells is consistent with our yeast two-hybrid screen result. Previous studies showed that the association of protein kinases with the Hsp90/cdc37 complex usually occurs through the catalytic kinase domain.^17^ Our results suggest that the pseudokinase MLKL interacts with Hsp90 through its C-terminal pseudokinase domain as well as N-terminal helical region.

### Hsp90 enhances MLKL-induced necroptosis

**Figure 2:**
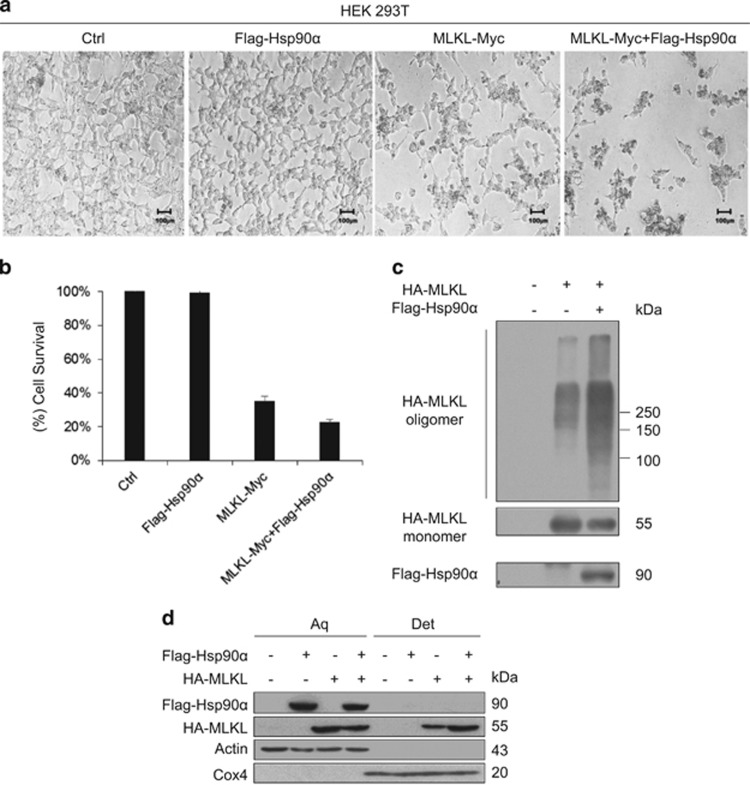
Coexpression of Hsp90 enhances MLKL-mediated necroptosis. (**a**) The 293T cells were transfected with vector, with Flag-Hsp90*α*, with MLKL-Myc alone, or with Flag-Hsp90*α* and MLKL-Myc. After 24 h of transfection, cell images were taken with a Nikon-TE2000 microscope. Scale bar, 100 *μ*m. (**b**) The 293T cells were transfected as in (**a**). After 24 h of transfection, cell death was quantified by propidium iodide (PI) staining. Cell death data are the means±S.D. of three independent experiments. (**c**) Hsp90*α* increases MLKL oligomerization. The 293T cells were transfected with the indicated plasmids. The cells were harvested 24 h after transfection, and non-reducing samples (without *β*-mercaptoethanol) of whole-cell lysate were analyzed by immunoblotting with anti-HA antibody. (**d**) Hsp90*α* increases the plasma membrane translocation of MLKL. The 293T cells were transfected with the indicated expression vectors. After 24 h of transfection, the cells were harvested and lysed in Triton X-114 lysis buffer and then separated into aqueous phase (Aq) and detergent phase (Det) as described in the experimental procedures. The samples were resolved and probed with the indicated antibodies. *β*-actin and Cox4 were used as loading controls for soluble protein and membrane protein, respectively

The molecular chaperone Hsp90 regulates the conformation and activity of many protein kinases. To investigate whether Hsp90 regulates MLKL function, we transiently transfected 293T cells with MLKL alone or with MLKL and Hsp90. Overexpression of MLKL in 293T cells induced necroptosis. Of interest, co-transfection of Hsp90 notably enhanced MLKL-induced necroptosis (Figures 2a and b). Next, we examined the effect of Hsp90 overexpression on the oligomerization and plasma membrane translocation of MLKL. As shown in Figure 2c, overexpressed HA-MLKL oligomerized in 293T cells. Coexpression of Flag-Hsp90*α* significantly increased the oligomerization of HA-MLKL. Following oligomerization, a fraction of overexpressed HA-MLKL translocated to the plasma membrane. In the presence of Flag-Hsp90*α*, the amount of HA-MLKL translocated to the plasma membrane was markedly increased (Figure 2d). We also observed that Hsp90 increased the N-terminal MLKL helical region MLKL 1–178 induced necroptosis in 293T cells (Supplementary Figure S1). The oligomerization and plasma membrane translocation of MLKL is essential for MLKL-mediated necroptosis. These results suggest that Hsp90 facilitates the oligomerization and plasma membrane translocation of MLKL and enhances MLKL-activated necroptosis.

### Hsp90 regulates MLKL and RIP3 stability

**Figure 3:**
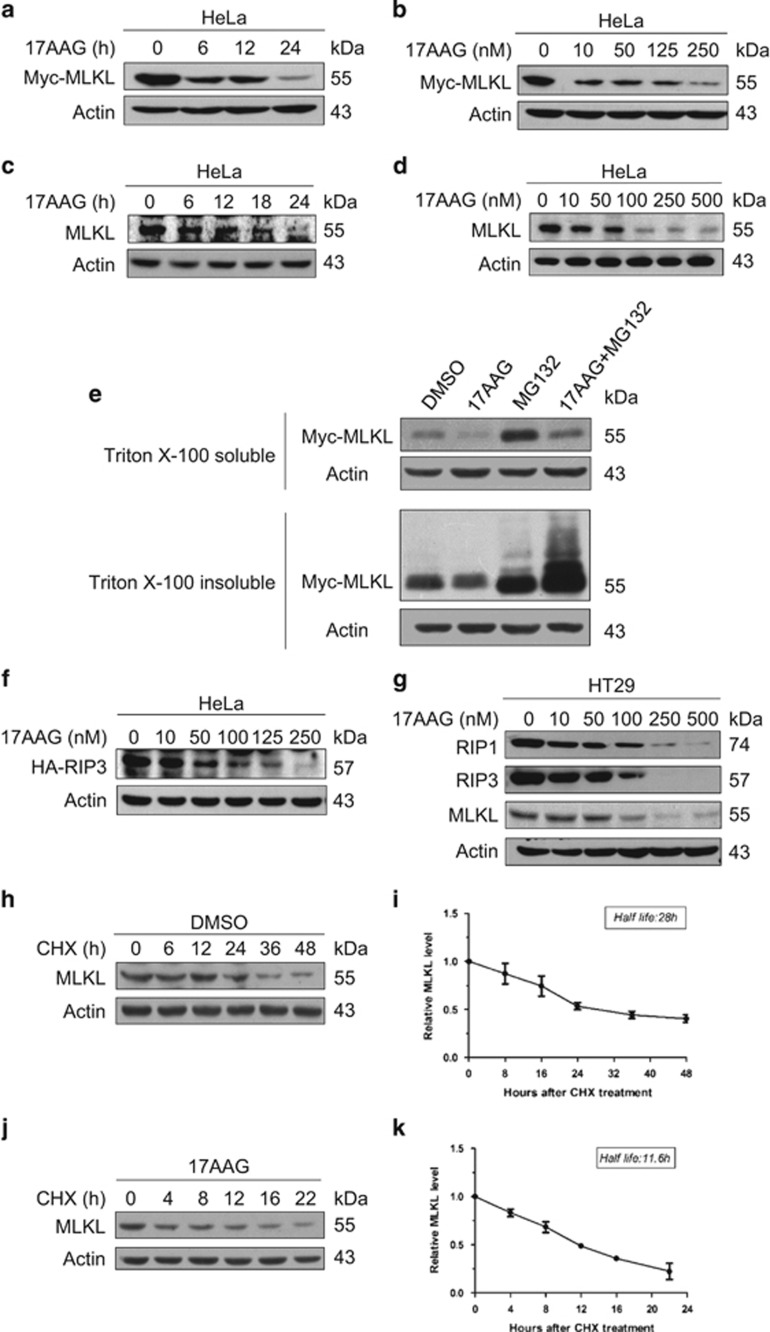
Hsp90 regulates MLKL and RIP3 stability. (**a**) 17AAG decreases the levels of ectopically expressed Myc-MLKL protein. HeLa cells were transfected with the Myc-MLKL plasmid. After 24 h of transfection, cells were treated with 250 nM 17AAG for the indicated times. Myc-MLKL protein levels were analyzed by western blotting with anti-Myc antibody. (**b**) HeLa cells transfected with Myc-MLKL were treated with the indicated concentrations of 17AAG for 24 h. Cell lysates were probed with anti-Myc antibody. (**c**) 17AAG reduces endogenous MLKL protein levels. HeLa cells were treated with 250 nM 17AAG for the indicated times. The cell lysates were resolved on SDS-PAGE and analyzed by immunoblotting with anti-MLKL-specific antibody. Actin was used as a loading control. (**d**) HeLa cells were treated with the indicated concentrations of 17AAG for 24 h. The levels of endogenous MLKL protein were analyzed by western blotting with anti-MLKL-specific antibody. Actin was the loading control. (**e**) 17AAG-induced degradation of MLKL is mediated by the proteasome pathway. HeLa cells transfected with Myc-MLKL were treated with or without 250 nM 17AAG in the presence or absence of 25 *μ*M MG132 for 8 h as indicated. MG132 was added 0.5 h prior to 17AAG treatment. Cells were lysed with Triton X-100 lysis buffer (1% Triton X-100). Insoluble precipitates were resolubilized with 2% SDS lysis buffer. Equal amounts of protein were analyzed by western blotting with anti-Myc antibody. (**f**) 17AAG affects exogenous RIP3 protein levels. HeLa cells transfected with HA-RIP3 were treated with increasing concentrations of 17AAG for 24 h. The levels of HA-RIP3 were determined by western blotting with anti-HA antibody. (**g**) The effect of 17AAG on endogenous RIP1/RIP3/MLKL protein levels. HT29 cells were treated with different concentrations of 17AAG for 24 h. Cell lysates were probed with anti-RIP1, anti-RIP3 and anti-MLKL-specific antibodies. Actin was the loading control. (**h**, **j**) 17AAG decreases the half-life of endogenous MLKL protein. HeLa cells were treated with 250 nM 17AAG for 12 h before the addition of CHX (25 *μ*g/ml). Cells were harvested at the indicated times after CHX addition, and the levels of MLKL protein were determined by immunoblotting with anti-MLKL-specific antibody. (**i**, **k**) Quantification of the experiments shown in (**h**) and (**j**). The values shown are obtained from three independent experiments and are normalized to the *β*-actin control

Hsp90 has been implicated in the stabilization of a number of kinases, including pp60v-src and Raf-1.^18, 19^ Loss of Hsp90 function results in their destabilization and rapid degradation by the ubiquitin–proteasome pathway. To examine whether Hsp90 may regulate MLKL stability, Hela cells transfected with Myc-MLKL were treated with the Hsp90-specific inhibitor 17-N-Allylamino-17-demethoxygeldanamycin (17AAG) for 0–24 h. As shown in Figure 3a, western blotting of cell extracts with anti-Myc antibody revealed that the level of Myc-MLKL was significantly decreased after 24 h of treatment with 17AAG. Next, we treated Hela cells transfected with Myc-MLKL for 24 h with increasing concentrations of 17AAG. 17AAG treatment reduced the amount of Myc-MLKL and the effect of 17AAG was concentration dependent (Figure 3b). To determine whether Hsp90 controls the stability of endogenous MLKL, Hela cells were treated with 250 nM 17AAG for different periods of time or with increasing concentrations of 17AAG for 24 h and subsequently analyzed for MLKL protein by immunoblotting with a specific antibody. 17AAG decreased the level of endogenous MLKL in a time- and concentration-dependent manner (Figures 3c and d). The proteasome inhibitor MG132 prevented the loss of MLKL protein and caused it to accumulate in a Triton X-100-insoluble cellular fraction (Figure 3e). A similar effect has been reported for Raf and PDK-1 previously.^20, 21^ This result suggests that a proteasome-mediated pathway degrades MLKL in the absence of Hsp90. Taken together, these results demonstrate the binding of Hsp90 to MLKL is critical for MLKL stabilization.

**Figure 4:**
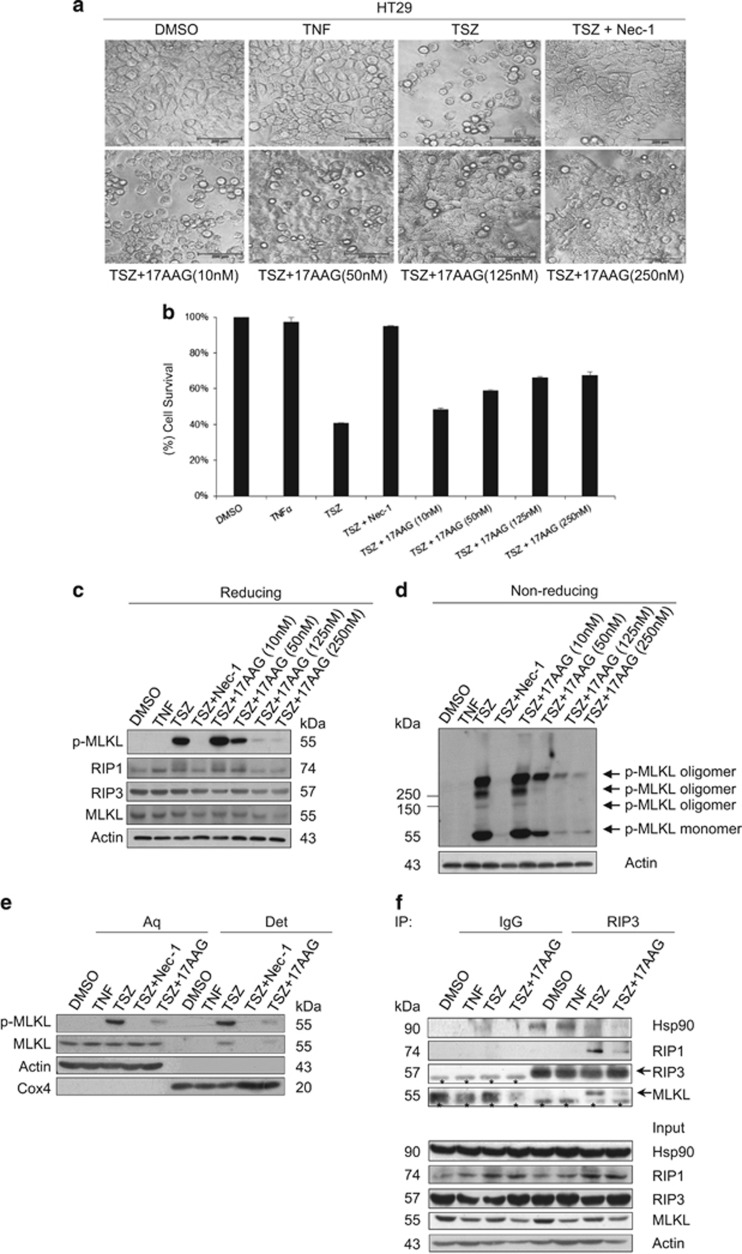
Hsp90 is required for TNF-triggered necroptosis. (**a**, **b**) 17AAG treatment inhibits TNF-induced necroptosis. HT29 cells were pretreated with or without different concentrations of 17AAG for 12 h, and then treated with the indicated stimuli for 24 h. Cell images were taken with a Nikon-TE2000 microscope (**a**). Scale bar, 200 *μ*m. Cell viability was determined by an MTS assay (**b**). Results are the means±S.D. of triplicate measurements. The final concentrations of 20 ng/ml TNF, 100 nM Smac mimetic, 20 *μ*M z-VAD and 10 *μ*M necrostatin-1 were used. T, TNF; S, Smac mimetic; Z, z-VAD; Nec-1, necrostatin-1. (**c**) 17AAG treatment reduces the phosphorylation of MLKL. HT29 cells were pretreated with or without different concentrations of 17AAG for 12 h, and then treated with the indicated stimuli for 8 h. The activation of MLKL was analyzed by immunoblotting with a T357/S358 phospho-specific MLKL antibody. Protein levels were detected by western blotting using anti-RIP1, anti-RIP3, anti-MLKL and anti-actin antibodies. (**d**) 17AAG inhibits TNF-induced oligomerization of phosphorylated MLKL. HT29 cells were treated as in (**c**). The cells were harvested and lysed, and non-reducing samples (without *β*-mercaptoethanol) of whole-cell lysate were analyzed by immunoblotting with a T357/S358 phospho-specific MLKL antibody. (**e**) 17AAG inhibits plasma membrane translocation of phosphorylated MLKL. HT29 cells were pretreated with or without 125 nM 17AAG for 12 h, and then treated with the indicated stimuli for 8 h. The cells were harvested and then separated into the aqueous phase (Aq) and detergent phase (Det) using Triton X-114 lysis buffer as described in the experimental procedures. The samples were analyzed by western blotting with the indicated antibodies. (**f**) The effect of 17AAG on TNF-induced necrosome formation. Ten-centimetre plates of HT29 cells were pretreated with or without 125 nM 17AAG for 12 h and then stimulated with TNF alone or TSZ for 8 h. Cells were then harvested and whole-cell extracts were immunoprecipitated with anti-RIP3 antibody or anti-IgG antibody and subsequently analyzed by western blotting for the indicated proteins. The asterisks denote nonspecific IgG bands

A previous study described that RIP1 is also an Hsp90-associated kinase. Disruption of Hsp90 function by the Hsp90 inhibitor geldanamycin (GA) resulted in the degradation of RIP1 and blockage of TNF-induced NF-*κ*B activation.^22^ We examined if Hsp90 also regulates the stability of the downstream RIP3 kinase. Figures 3f and g show that the expression levels of both overexpressed and endogenous RIP3 were greatly reduced in 17AAG-treated cells, suggesting that Hsp90 also controls RIP3 stability. Supporting this notion, we found that Hsp90 interacted with both ectopically expressed and endogenous RIP3 (Figures 1g and 4f).

Next, we determined the half-life of MLKL after 17AAG treatment. As Figure 3h shown, the half-life of MLKL in control cells was >28 h. 17AAG preincubation markedly destabilized MLKL and reduced the half-life of MLKL to 12 h (Figure 3j).

### Hsp90 is essential for TNF-induced necroptosis

To assess the cellular role of Hsp90 in TNF-induced necroptosis, we tested the effect of the Hsp90-specific inhibitor 17AAG on TNF-induced necroptosis. The human colorectal cancer HT29 cells were left untreated or treated with the combination of TNF (T), Smac mimetic (S) and the pan-caspase inhibitor z-VAD-fmk (Z). HT29 cells underwent necroptosis in response to TSZ stimulation. As expected, TSZ-induced necroptosis was blocked by the RIP1 kinase inhibitor, necrostatin-1 (Nec-1). Of interest, 17AAG pretreatment inhibited TSZ-induced necroptosis in a concentration-dependent manner (Figures 4a and b, Supplementary Figures S3 and S4). Next, we examined the phosphorylation of MLKL by western blotting using a phospho-MLKL-specific antibody. As shown in Figure 4c, TSZ induced robust MLKL phosphorylation, which was blocked by Nec-1. Consistent with inhibition of necroptosis, >125 nM 17AAG pretreatment strongly reduced phosphorylation of MLKL. 17AAG also severely inhibited oligomerization and plasma membrane translocation of MLKL following TSZ stimulation in HT29 cells (Figures 4d and e). Of note, in this experiment while high concentration of 17AAG (250 nM) resulted in degradation of RIP1, RIP3 and MLKL, and strongly suppressed TNF-induced necroptosis; low concentration of 17AAG (125 nM) significantly inhibited phosphorylation of MLKL, RIP1 and possibly RIP3 without markedly affecting their protein levels. The phosphorylation of RIP1 was represented by the RIP1 bands shift in SDS-PAGE (Figure 4c). These results, together with the results obtained in the MLKL regulation study in 293T cells (Figure 2), suggest that in addition to controlling the stability of RIP1, RIP3 and MLKL, Hsp90 also regulates their activity.

As the RIP1/RIP3/MLKL necrosome is a critical signaling platform for MLKL activation and TNF-induced necroptosis, we then examined if Hsp90 regulates necrosome assembly. We performed a coimmunoprecipitation assay with anti-RIP3 antibody. As shown in Figure 4f, TSZ stimulated the binding of RIP1 and MLKL to RIP3. 17AAG preincubation disrupted the Hsp90-RIP1-RIP3-MLKL complex and severely inhibited the formation of necrosome. Of interest, Hsp90 formed complex with RIP3 and probably with RIP1 and MLKL in HT29 cells before TSZ stimulation. Following TSZ stimulation, a significant amount of Hsp90 dissociated from the necrosome after the assembly and maturation of necrosome (Figure 4f). Taken together, these results suggest that Hsp90 has a critical role in the formation of necrosome and is required for TNF-induced necroptosis.

## Discussion

In this study, we have demonstrated that the molecular chaperone Hsp90 is a novel MLKL-interacting protein. We have shown that Hsp90 is essential for the stabilization of MLKL. In addition, we find that Hsp90 is required for RIP3 stability. We show that complex formation with Hsp90 is a prerequisite for the activation of RIP3 and MLKL and for the formation of necrosome in response to TNF stimulation. Disruption of Hsp90 function induces the degradation of MLKL and RIP3 and inhibits TNF-induced necroptosis. Our results demonstrate that Hsp90 regulates MLKL and RIP3 stability and is essential for TNF-induced necroptosis. These findings, together with two recent studies reporting that Hsp90 controls RIP3 function/stability^23, 24^ and an earlier study describing RIP1 is an Hsp90 kinase client,^22^ reveal that Hsp90 regulates the function of all the three components of the RIP1/RIP3/MLKL cascade, which mediates TNF-triggered necroptosis. Intriguingly, previous reports suggested that pretreatment with the Hsp90 inhibitor GA protects Jurkat cells from DD receptor-induced necroptosis and disruption of Hsp90 function reverts TNFR1-induced necroptosis to apoptosis, although the underling mechanisms were not understood well at that time.^2, 25^

A significant number of protein kinases form complexes with Hsp90 as a prerequisite for activation and/or assembly into functional complexes. Hsp90 contributes to the stabilization and maturation of these kinases and maintains them in their functionally active conformations.^17^ The requirement of Hsp90 for the function of MLKL and RIP3 provides new evidences for the control of kinase activities by Hsp90. We demonstrated that Hsp90 is essential for the stabilization of MLKL and RIP3 proteins. It is conceivable that regulation of MLKL and RIP3 stability is an important step in the formation of necrosome in response to TNF stimulation. By directly modulating the stability of RIP1, RIP3 and MLKL, Hsp90 may hence control the signal-transducing capacity of these kinases. In addition to regulating their stability, Hsp90 may act as a molecular chaperone to assist the folding and maturation of RIP1, RIP3 and MLKL. Hsp90 has been shown to help to maintain the steroid receptor in a conformation competent for the binding of the hormone.^17^ It is tempting to speculate that Hsp90 may assist MLKL to adopt multiple conformational states that facilitate or inhibit interactions with regulatory partners such as RIP3. Alternatively, Hsp90 could regulate the susceptibility to post-translational modification of MLKL by RIP3 and facilitate the conformational transition of MLKL from ‘inactive state' to ‘activated state'. Consistent with this idea, we found that overexpression of Hsp90 enhances the oligomerization of MLKL in 293T cells (Figure 2c). Intriguingly, in line with our finding, overexpression of the Hsp90 co-chaperone Cdc37 increases the oligomerization and activation of IRAK-1 in 293T cells.^26^ Hsp90 may also facilitate necrosome assembly. We observed that Hsp90 associates with the RIP1-RIP3-MLKL complex (Figure 4f). It seems that the complex formation of Hsp90 with RIP1-RIP3-MLKL is required for necrosome assembly, as disrupting the Hsp90-RIP1-RIP3-MLKL complex with 17AAG strongly inhibits TNF-stimulated necrosome formation (Figure 4f). Hsp90 may not recruit RIP3 and MLKL directly to RIP1; it may instead keep RIP3 and MLKL in a conformation able to respond to appropriate stimuli. Hsp90 could also have a role in the translocation of necrosome from the cytoplasm to the plasma membrane. It has been demonstrated that Hsp90/Cdc37 associates with the IKK complex and is required for trafficking of IKK from the cytoplasm to the membrane. Inhibition of Hsp90 prevented the recruitment of the IKK complex to TNFR1.^27^ In addition, it was shown that Hsp90 is essential for the Ras-dependent trafficking of Raf kinase from the cytosol to the plasma membrane, and GA treatment blocked Raf translocation.^18, 28^ Our results demonstrated that 17AAG preincubation inhibits TNF-induced translocation of MLKL to the plasma membrane (Figure 4e), suggesting that Hsp90 has an important role in trafficking of the necrosome from the cytoplasm to the membrane.

Hsp90 has been shown to be released from its clients upon activation, for example, in the case of steroid receptor, Hsp90 dissociates from the receptor upon ligand binding;^29^ and in the case of CDK4, Hsp90 is released from the active CDK4/cyclin D complex before it translocates into the nucleus to promote cell cycle progression.^30^ We observed that a significant amount of Hsp90 is released from the RIP1-RIP3-MLKL complex following necrosome formation (Figure 4f). This result suggests that although Hsp90 is required for TNF-induced necrosome assembly, it might not be a part of the final necrosome complex.

In conclusion, the results we have presented here and those reported by earlier studies^22, 23, 24^ provide a molecular basis for the inhibitory effect of Hsp90 inhibitors on TNF-induced necroptosis. Previous studies suggested that IKK kinases are additional protein kinases involved in TNFR1 signaling that also depend on Hsp90 for their functions.^27, 31^ Our findings together with those studies demonstrate that Hsp90 modulates the activity of multiple components of TNFR1 signaling pathways (for example, RIP1, RIP3, MLKL and IKK kinases) and reveal that Hsp90 has crucial roles in TNF-mediated activation of NF-*κ*B and necroptosis. Necroptosis has been implicated in mediating multiple human inflammatory diseases.^1^ Pharmacological inhibitors of Hsp90 could be potential therapeutic agents for treatment of human diseases characterized by necrosis and inflammation.

## Materials and Methods

### Cell culture and reagents

Human HT29, HEK293T and HeLa cells were cultured in DMEM (Life Technologies, Carlsbad, CA, USA) supplemented with 10% fetal bovine serum (Hyclone, Logan, UT, USA) and 100 *μ*g/ml penicillin and streptomycin at 37 °C in a humidified incubator containing 5% CO_2_. The following reagents were used: Hsp90 inhibitor 17AAG (Selleckchem, Huston, TX, USA, S1141); z-VAD-fmk (ApexBio, Huston, TX, USA, A1902); Triton X-114 (Sigma-Aldrich, St. Louis, MO, USA); anti-HA (sc-805), anti-Myc (sc-40), anti-RIP3 (sc-374639) (Santa Cruz Biotechnology, Santa Cruz, CA, USA); anti-Hsp90 (ab178854), anti-phospho-MLKL (ab187091) (Abcam, Cambridge, MA, USA); anti-MLKL (Merck Millipore, Billerica, MA, USA, MABC604); anti-Flag M2 (Sigma-Aldrich); anti-RIP1 (BD Pharmingen, Franklin Lakes, NJ, USA, 51-6559GR). HRP-conjugated goat anti-mouse and anti-rabbit were from Thermo Scientific, Waltham, MA, USA. The smac mimetic SM-164 was a kind gift of Dr. Shaomeng Wang (University of Michigan Comprehensive Cancer Center, Ann Arbor, MI, USA).

### Yeast two-hybrid screen

The N-terminal region (1–178aa) of MLKL was used as a bait to screen a human bone marrow MATCHmaker cDNA library cloned into pACT2 (Clontech, Mountain View, CA, USA) according to the manufacturer's instructions. In brief, the *Saccharomyces cerevisiae* reporter strain AH109 was sequentially transformed with the plasmid pGBKT7-MLKL (1–178) and the library. The yeast transformants were selected by growth on SD/-His/-Leu/-Trp media. Approximately 9 × 10^6^ transformants were screened. Positive clones were confirmed using a *β*-galactosidase activity assay. Plasmids harboring interacting cDNAs were isolated from yeast and transformed into *E.coli* and purified and subjected to sequencing and NCBI BLAST analysis.

### Expression constructs and cell transfection

Full-length Hsp90 and MLKL were amplified by PCR and subcloned into pCMV-Myc/HA vector (Clontech). FLAG-tagged Hsp90 was PCR amplified and cloned into pCMV-Tag2 vector (Stratagene, Santa Clara, CA, USA). The MLKL deletion mutants were constructed by PCR and inserted into the pCMV-Myc vector. All constructs were verified by DNA sequencing. Cell transient transfections were carried out using a standard calcium phosphate method.

### Immunoblotting and immunoprecipitation

Cells at 90% confluence were washed once with PBS (PH7.4) and harvested by scraping. Cells were then lysed in cell lysis buffer containing 20 mM Tris-HCl, pH 7.5, 150 mM NaCl, 10 mM NaF, 20 mM *β*-glycerophosphate, 1 mM sodium orthovanadate, 1 mM PMSF, 10 *μ*g/ml leupeptin, 2 *μ*g/ml aprotinin, 1% Triton X-100 and 1 mM EDTA for immunoprecipitation. Protein concentration was determined by the BCA assay (Thermo Pierce, Waltham, MA, USA) and immunocomplexes were resolved by SDS-PAGE and probed with specific antibodies. The Triton X-100 insoluble proteins were solubilized with SDS lysis buffer (2% SDS, 100 mM DTT, 80 mM Tris-HCl, pH 6.8 and 10% glycerol) and equal amounts of protein were analyzed by western blotting.

### Protein half-life assay

HeLa cells were treated with 25 *μ*g/ml cycloheximide (CHX) (Sigma-Aldrich) for different times. At each time point after CHX treatment, cells were harvested and lysed with Triton X-100 lysis buffer, and protein levels were detected by western blotting using specific anti-MLKL and anti-actin antibodies. MLKL protein levels relative to *β*-actin control were quantified from three independent experiments.

### Fractionation by phase separation

Cell were lysed in Triton X-114 lysis buffer (20 mM HEPES, pH 7.4, 150 mM NaCl, 2% Triton X-114, 1 mM PMSF, 10 *μ*g/ml leupeptin and 2 *μ*g/ml aprotinin) and separated into the aqueous faction (Aq) and the detergent fraction (Det) as described previously.^14^

### Cell death assays

For 293T cells transfected with MLKL-expressing constructs, cell death was analyzed by propidium iodide (PI) staining (1 *μ*g/ml). Necrotic cells (PI-positive, red nuclei) were counted in comparison with total population (*n*=300 cells). Each assay was repeated a minimum of three times. For HT29 cells treated with necroptotic stimulus, cell viability was determined by an MTS assay using Cell Titer 96 Aqueous One Solution Cell Proliferation Assay kit (G3580, Promega, Madison, WI, USA) according to the manufacturer's instruction.

## Abbreviations

17AAG: 17-*N*-allylamino-17-demethoxygeldanamycin
DD: death domain
Hsp90: heat shock protein 90
MLKL: mixed lineage kinase domain-like
Nec-1: necrostatin-1
RIP1: receptor-interacting protein kinase 1
RIP3: receptor-interacting protein kinase 3
Smac: second mitochondria-derived activator of caspase
TNF: tumor necrosis factor
z-VAD: Z-VAD-fmk
