# Supplementary material for: Hsp90 modulates the stability of MLKL and is required for TNF-induced necroptosis
Source: Cell Death Dis. 2016 Feb 11;7(2):e2089–. doi: 10.1038/cddis.2015.390 (PMC4849146; doi:10.1038/cddis.2015.390)
Supplement: Supplementary Information [file cddis2015390x1.pdf]

Supplementary Figure 1

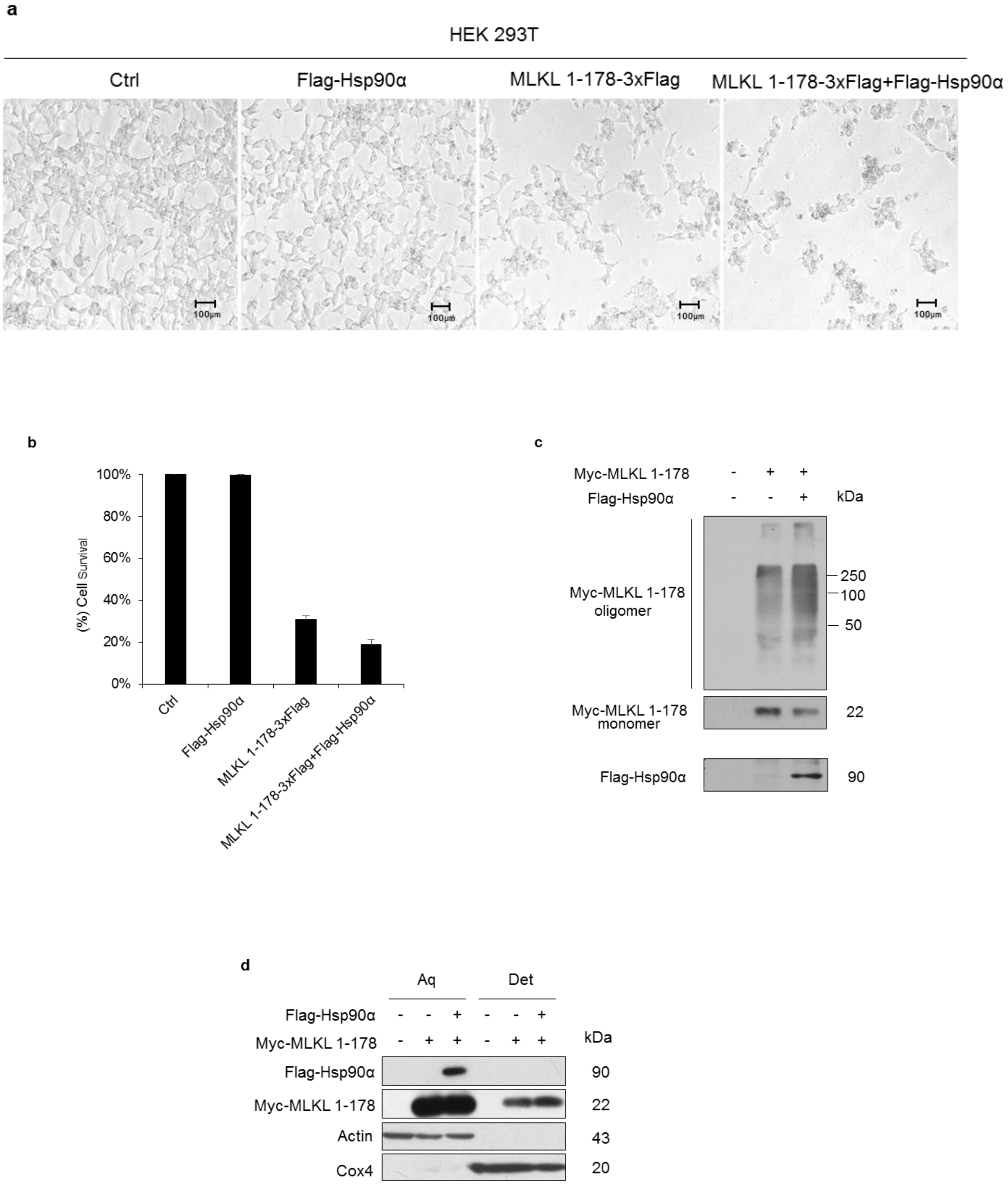

Supplementary Figure 2

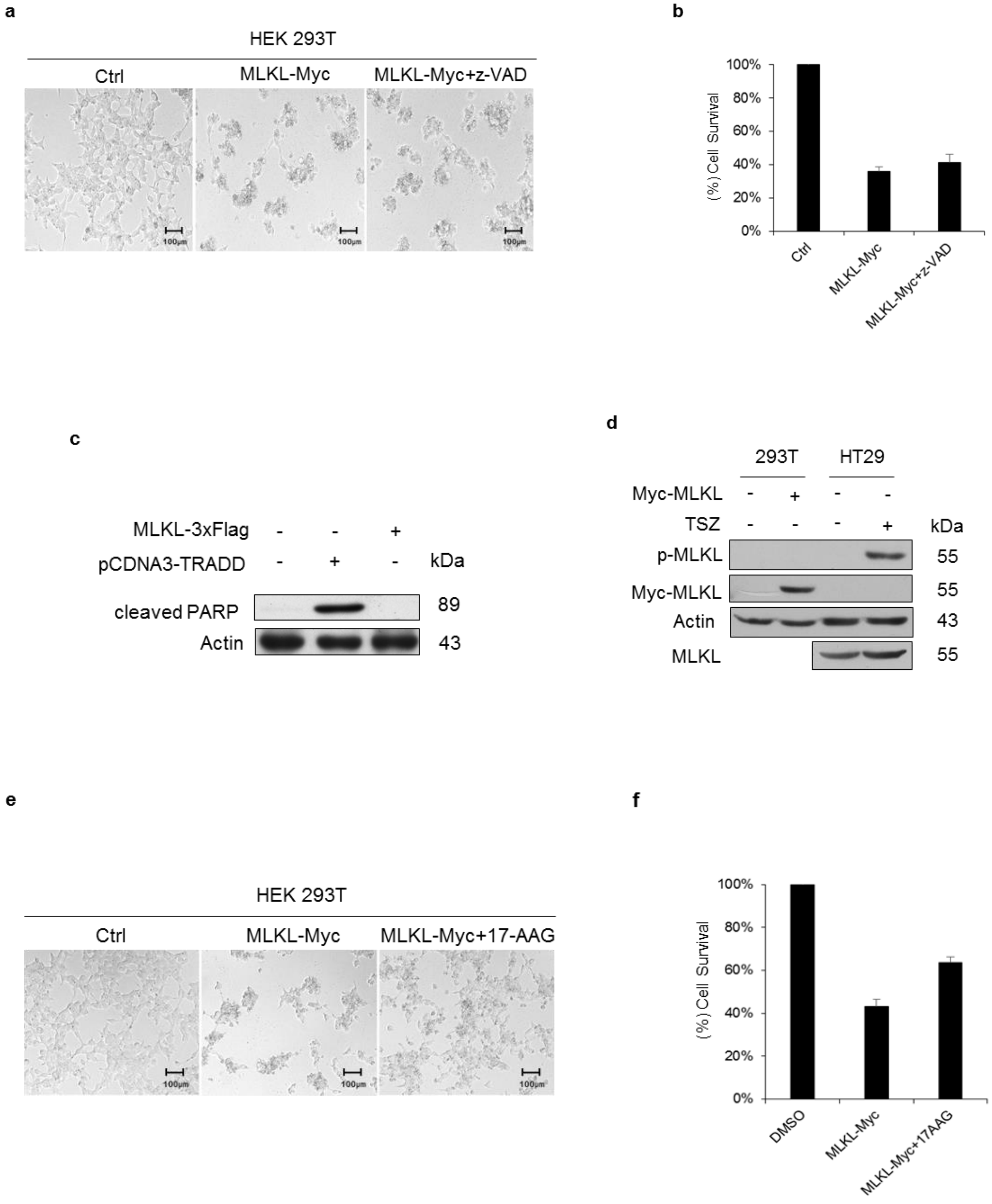

Supplementary Figure 3

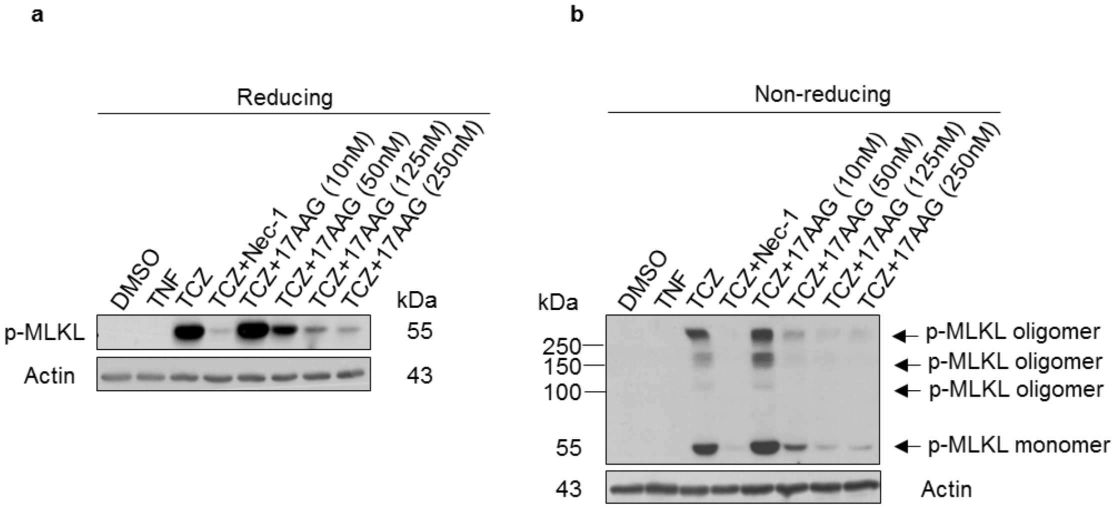

Supplementary Figure 4

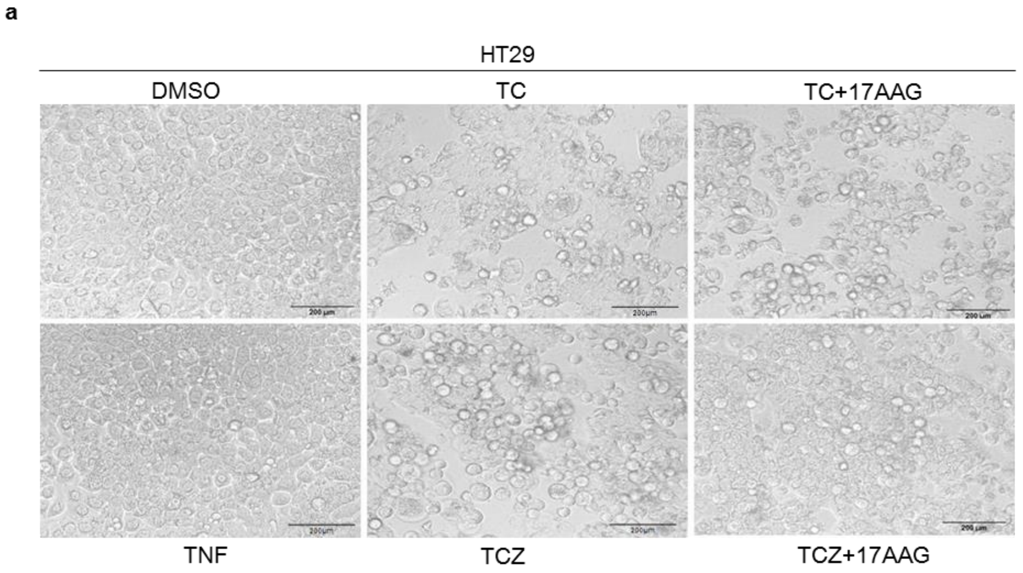

b

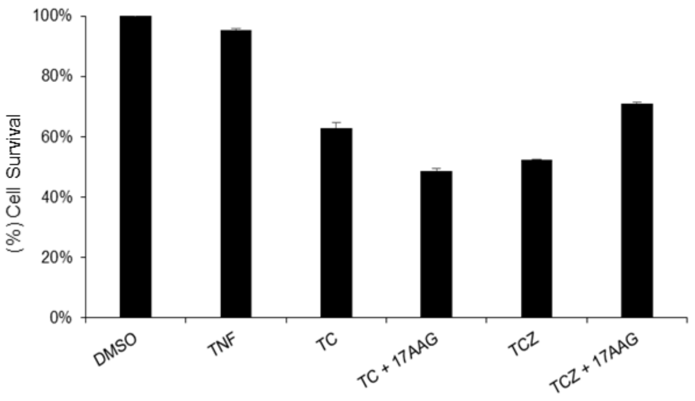

Supplementary Figure Legends

Supplementary Figure 1 Hsp90 enhances the MLKL helical region-mediated necroptosis. (a) The 293T cells were transfected with vector, with Flag-Hsp90α, with MLKL 1-178-3×Flag alone, or with Flag-Hsp90α and MLKL 1-178-3×Flag. 24hr post-transfection, cell images were taken with a Nikon-TE2000 microscope. Scale bar, 100μm. (b) The 293T cells were transfected as in (a). Cell death was quantified by propidium iodide (PI) staining. Cell death data are the means ± SD of three independent experiments. (c) Hsp90α increases MLKL 1-178 oligomerization. The 293T cells were transfected with Myc-MLKL 1-178 alone, or with Flag-Hsp90α and Myc-MLKL 1-178. The cells were harvested and non-reducing samples (without β-mercaptoethanol) of whole cell lysate were analyzed by immunoblotting with anti-Myc antibody. (d) Hsp90α increases the plasma membrane translocation of MLKL 1-178. The 293T cells were transfected with the indicated expression vectors. 24hr after transfection, the cells were harvested and lysed in Triton X-114 lysis buffer and then separated into aqueous phase (Aq) and detergent phase (Det) and probed with the indicated antibodies. β-actin and Cox4 were used as loading controls.

Supplementary Figure 2 Overexpression of MLKL-Myc induced cell death in 293T cells is caspase-independent. (a) The 293T cells were transfected with MLKL-Myc in the absence or presence of 20μM z-VAD-fmk. 24hr post-transfection,

cell images were taken with a Nikon-TE2000 microscope. Scale bar, 100μm. (b) The 293T cells were transfected as in (a). Cell death was quantified by propidium iodide (PI) staining. Cell death data are the means ± SD of three independent experiments. (c) The 293T cells were transfected with pcDNA3-TRADD or MLKL-3×Flag. Cell lysate were probed with anti-cleaved PARP antibodies. Actin was the loading control. (d) Overexpression of MLKL in 293T cells does not cause the phosphorylation of MLKL. The 293T cells were transfected with Myc-MLKL. Cell lysate were probed with a T357/S358 phospho-specific MLKL antibody. HT29 cell lysate stimulated with TSZ were used as a positive control. (e, f) The 293T cells were pre-incubated with DMSO or 250 nM 17AAG for 12hr, then transfected with MLKL-Myc. 24hr post-transfection, cell images were taken with a Nikon-TE2000 microscope (e). Cell death was quantified by propidium iodide (PI) staining (f). Cell death data are the means ± SD of three independent experiments.

Supplementary Figure 3 17AAG inhibits TCZ-induced phosphorylation and oligomerization of MLKL. (a) 17AAG reduces TCZ-induced phosphorylation of MLKL. HT29 cells were pretreated with or without different concentrations of 17AAG for 12hr, and then treated with the indicated stimuli for 8 hr. The phosphorylation of MLKL was analyzed by Western blot with a T357/S358 phospho-specific MLKL antibody. Actin was the loading control. The final concentrations of 20ng/ml TNF, 20μg/ml cycloheximide, 20μM z-VAD, and 10μM necrostatin-1 were used. T, TNF; C, cycloheximide; Z, z-VAD; Nec-1, necrostatin-1. (b) 17AAG inhibits TCZ-induced oligomerization of phosphorylated MLKL. HT29 cells were treated as in (a). The cells were harvested and lysed, and non-reducing samples (without β-mercaptoethanol) of whole cell extract were analyzed by Western blot with a T357/S358 phospho-specific MLKL antibody.

Supplementary Figure 4 17AAG inhibits TNF-induced necroptosis, but not apoptosis. (a, b) HT29 cells were pretreated with or without 125nM 17AAG for 12hr, and then treated with the indicated apoptotic stimulus (TC) or necroptotic stimulus

(TCZ) for 24 hr. Cell images were taken with a Nikon-TE2000 microscope (a). Scale bar, 200μm. Cell viability was determined by an MTS assay (b). Results are the means ± SD of triplicate measurements.
